# Negative anti-neutrophil cytoplasm antibody at switch to maintenance therapy is associated with a reduced risk of relapse

**DOI:** 10.1186/s13075-017-1321-1

**Published:** 2017-06-07

**Authors:** Matthew David Morgan, Matthew Szeto, Michael Walsh, David Jayne, Kerstin Westman, Niels Rasmussen, Thomas F. Hiemstra, Oliver Flossmann, Annelies Berden, Peter Höglund, Lorraine Harper, on behalf of the European Vasculitis Society

**Affiliations:** 1 Renal Immunobiology, Institute of Clinical Sciences, College of Medical and Dental Sciences, University of Birmingham, Birmingham, UK; 2 Departments of Medicine and Clinical Epidemiology and Biostatistics, McMaster University, Hamilton, Canada; 3 Population Health Research Institute, Hamilton Health Sciences/McMaster University, Hamilton, Canada; 4 Department of Medicine, Addenbrooke’s Hospital, University of Cambridge, Cambridge, UK; 5 Department of Nephrology, Skane University Hospital Malmö, Lund University, Lund, Sweden; 6 Department of Autoimmune Serology, Statens Seruminstitut, Copenhagen, Denmark; 7 School of Clinical Medicine, University of Cambridge and Cambridge Clinical Trials Unit, Addenbrooke’s Hospital, Cambridge, UK; 8 Renal Department, Royal Berkshire Hospital, Reading, UK; 9 Department of Internal Medicine, Leiden University Medical Center, Leiden, The Netherlands; 10 Competence Centre for Clinical Research, Skane University Hospital, Lund, Sweden; 11 Queen Elizabeth Hospital Birmingham, Area 5, Level 7, Mindelsohn Way, Edgbaston, Birmingham, B15 2WB UK

**Keywords:** ANCA, Vasculitis, Relapse, Treatment, ANCA-associated vasculitis, Clinical trial, Prognostic factors

## Abstract

**Background:**

Relapse of disease is frequent in anti-neutrophil cytoplasm antibody (ANCA)-associated vasculitis (AAV). It is unclear whether persistent ANCA when starting maintenance therapy increases the risk of relapse. We examined the association between ANCA status and relapse in two randomised controlled trials.

**Methods:**

ANCA-positive patients in two trials, CYCLOPS and IMPROVE, were switched from cyclophosphamide to maintenance therapy after achieving clinical remission. We classified patients as being either ANCA-positive or ANCA-negative at the time they started maintenance therapy. We compared the risk of relapse in ANCA-positive and ANCA-negative patients.

**Results:**

Of 252 patients included, 102 (40%) experienced at least one relapse during the follow-up period. At the time of the switch from induction to maintenance therapy, 111 were ANCA-positive, of whom 55 (50%) relapsed, compared to 141 patients who were ANCA-negative, of whom 47 (33%) relapsed. In multivariable time-to-event analysis, a reduced risk of relapse was associated with having become ANCA-negative at the time of switching to maintenance therapy (hazard ratio 0.63, 95% confidence interval 0.42–0.95; *p* = 0.026). In addition, initial proteinase 3 (PR3)-ANCA, younger age, lower serum creatinine, pulsed cyclophosphamide for remission induction, and mycophenolate mofetil for remission maintenance were all associated with an increased risk of relapse.

**Conclusions:**

Becoming ANCA-negative before the switch to maintenance is associated with a reduced risk of relapse.

**Trial registration:**

CYCLOPS: ClinicalTrials.gov, NCT00430105. Registered retrospectively on 31 January 2007. IMPROVE: ClinicalTrials.gov, NCT00307645. Registered retrospectively on 27 March 2006.

## Background

Granulomatosis with polyangiitis (GPA) and microscopic polyangiitis (MPA) are potentially life-threatening inflammatory diseases. Whereas historically they were almost uniformly fatal, 5-year survival is now 78% but is often characterised by frequent relapses which are associated with significant morbidity and mortality [1–5].

Previous studies have identified several factors associated with an increased risk of relapse including the presence of circulating proteinase 3 (PR3)-specific, as opposed to myeloperoxidase (MPO)-specific, anti-neutrophil cytoplasm antibodies (ANCA) at the time of disease diagnosis [6, 7]. The role of ANCA testing after diagnosis in predicting relapse has been the subject of many previous studies, although a recent meta-analysis concluded that persistence or a rise in ANCA titre (irrespective of specificity) during remission was only modestly predictive of relapse [8].

Previous studies suggested that the persistence of PR3-ANCA, but not MPO-ANCA, at the time of discontinuing cyclophosphamide and switching to maintenance therapy is associated with an increased risk of relapse [7, 9]. We used long-term follow-up data from two of the previous European Vasculitis Study Group (EUVAS) clinical trials, CYCLOPS and IMPROVE, to identify whether persistent ANCA positivity at the time of discontinuing cyclophosphamide and switching to maintenance therapy is associated with an increased risk of relapse in patients with moderate-to-severe ANCA-associated vasculitis (AAV) treated with cyclophosphamide as initial remission induction therapy [10, 11].

## Methods

### Patient population and trial protocols

The trial databases of two EUVAS trials (CYCLOPS [10] and IMPROVE [11]) and long-term follow-up data from the CYCLOPS trial cohort were examined [12].

Briefly, CYCLOPS compared pulse cyclophosphamide versus daily oral cyclophosphamide for inducing remission in 149 patients with newly diagnosed generalised AAV from 42 centres in 13 European countries and Mexico between 1998 and 2002. Patients received cyclophosphamide until 3 months after achieving remission (maximum 12 months) and then converted to azathioprine for maintenance therapy. All patients received prednisolone, initially at 1 mg/kg body weight per day tapered to 12.5 mg per day at the end of month 3 and to 5 mg per day by 18 months.

IMPROVE compared mycophenolate mofetil (MMF) to azathioprine for maintenance of remission in AAV following remission induction in 156 patients with newly diagnosed AAV from 42 centres in 11 European countries between 2002 and 2004. Patients received either daily oral or pulsed cyclophosphamide according to investigator preference until remission or a maximum of 6 months following which they were randomised to azathioprine or MMF maintenance therapy. Both azathioprine and MMF were withdrawn at 42 months. Prednisolone was initially given at 1 mg/kg/day tapering to 5 mg/day after 12 months and discontinued at 24 months [11].

For this post-hoc study, patients who were ANCA-positive at trial entry and achieved remission following induction therapy with cyclophosphamide were included. Cases for which the duration of cyclophosphamide therapy and the time of switching to maintenance therapy could not be determined were excluded.

### Data collection

Patients in the CYCLOPS trial were initially followed-up for 18 months. Subsequently, long-term follow-up data were collected using physician-completed standardised data abstraction forms [12]. The following data were collected and used in this study: age and sex at diagnosis, ANCA specificity by enzyme linked immunosorbent assay (ELISA) at diagnosis and whether positive or negative by ELISA at the switch to maintenance therapy, time to clinical remission (as defined in the original trial protocols: the complete absence of clinical disease activity using the Birmingham Vasculitis Activity Score (BVAS) item list and supported by a normal serum C-reactive protein (CRP) concentration [13, 14]) and conversion to maintenance therapy, serum creatinine concentration at diagnosis, clinical diagnosis (GPA vs MPA/renal limited vasculitis), induction cyclophosphamide regime (pulse or daily oral cyclophosphamide), and initial maintenance therapy received. All ANCA assays were performed in local centres according to local protocols and reported as present or absent according to local upper limits of normal. Patients who were positive for both PR3- and MPO-ANCA on ELISA were classified as PR3-ANCA as previous studies have suggested that the presence or absence of PR3-ANCA is a significant predictor of relapse [15, 16].

Renal limited vasculitis was included with MPA based on findings of previous studies [8, 17].

The primary outcome for this study was first relapse. Relapses were defined in the trial protocols as a new occurrence or re-occurrence of major or minor organ involvement attributable to active vasculitis supported by exacerbation of at least two constitutional symptoms and a rise in CRP [13, 14].

### Statistical analyses

Categorical data are summarised as frequencies (%) and compared using Fisher’s exact test. Continuous measurements are expressed as medians (25^th^ to 75^th^ percentile) and compared using Mann-Whitey *U* tests. Multivariate binary logistic regression analysis was used to assess the independence of factors associated with remaining ANCA-positive or becoming ANCA-negative and is reported as the hazard ratio (HR) with 95% confidence interval (CI).

Relapse-free survival time was calculated from the point of remission to first relapse and censored for death or end of follow-up. Univariate Cox regression survival analysis was performed and reported as HR with 95% CI. Multivariable survival analysis was performed using Cox regression models containing all exposures listed above except clinical diagnosis. Clinical diagnosis was excluded from the multivariable models since PR3-ANCA specificity has previously been shown to be a better predictor of relapse than disease phenotype [18]. Confirmation that all covariates included in the Cox regression survival analyses met the proportional hazards assumption was assessed graphically using log (–log) survival curves and plotting partial residuals against time. To determine whether the risk of effect of ANCA status at time to switching to maintenance therapy on risk of relapse was similar for both MPO-ANCA and PR3-ANCA, a Cox regression model was constructed including both initial specificity, ANCA status at switch, and an interaction term between the two. The Nagelkerke pseudo *R* ^2^ value for the goodness-of-fit was calculated for two multivariable models, one including all covariates and a second with ANCA status at switch to maintenance therapy removed [19]. Statistical significance was defined as a *p* value less than 0.05. All analyses were carried out using IBM SPSS for Windows version 20.

## Results

### Patients

Two hundred and fifty-two patients met the entry criteria for this study. The median length of follow-up was 4 (3.5–4.4) years with a total of 956 patient-years of follow-up. One hundred and two patients experienced at least one relapse during follow-up and 11 died without experiencing a relapse. Other demographic and clinical data, including ANCA status, is shown in Table 1.

**Table 1:** Baseline characteristics of the patients and the associations between patient characteristics and ANCA status at switching to maintenance therapy. *p* refers to the difference between the ANCA +ve and ANCA-ve status at switch to maintenance therapy.

	All patients	ANCA –ve	ANCA + ve	*p*
*n* (%)	252	141 (56%)	111 (44%)	0.36
Male Female	158 94	92 49	66 45	
Age (years)	59 (47–67)	62 (50–68)	54 (42–66)	0.009
Creatinine at entry (μmol/L)	177 (108–239)	185 (126–327)	151 (97–258)	0.007
Creatinine strata (μmol/L)				0.077
< 100 101–200 > 200	54 90 108	24 49 68	30 41 40	
ANCA specificity at entry				0.7
MPO PR3	107 145	58 83	49 62	
Diagnosis				0.45
MPA GPA	118 134	63 78	55 56	
Time to remission (months)	3 (2.7–4.1)	3 (2–3.7)	3.2 (3–4.5)	0.001
Cyclophosphamide				0.53
Continuous Pulse	117 135	68 73	49 62	
Maintenance therapy				0.093
AZA MMF Other	175 73 4	103 34 4	72 39 0	
Relapse:no-relapse	102:150	47:94	55:56	0.025

*ANCA* anti-neutrophil cytoplasm antibodies, *AZA* azathioprine, *GPA* granulomatosis with polyangiitis, *MMF* mycophenolate mofetil, *MPA* microscopic polyangiitis, *MPO* myeloperoxidase, *PR3* proteinase 3

The associations between patient characteristics and ANCA status at the switch to maintenance therapy are shown in Table 1. In a multivariate binary logistic regression analysis patients who remained ANCA-positive were younger (HR 0.79 (0.65–0.97) per decade; *p* = 0.02), had lower serum creatinine at entry (HR 0.89 (0.81–0.98) per 50 μmol/L; *p* = 0.013), and took longer to achieve remission than those who became ANCA-negative (HR 1.29 (1.08–1.54) per month; *p* = 0.005). There was no difference in initial induction regime or whether patients were more likely to subsequently receive MMF or azathioprine as remission maintenance agents.

Univariate Cox regression survival analysis confirmed that remaining ANCA-positive at the time of switching to maintenance therapy was associated with an increased risk of relapse (Table 2). The sensitivity, specificity, and positive and negative predictive values of positive ANCA for relapse and negative ANCA for no relapse are shown in Table 3.

**Table 2:** Univariate survival analysis of factors potentially associated with the risk of relapse during follow-up

Variable	Hazard ratio (95% CI)	*p*
ANCA at switching to maintenance therapy		
ANCA-positive	1	0.004
ANCA-negative	0.57 (0.38–0.84)	
ANCA at trial entry		
MPO-ANCA	1	0.002
PR3-ANCA	1.99 (1.30–3.04)	
Patient age (per decade)	0.79 (0.70–0.90)	<0.001
Serum creatinine at entry (per 50 μmol/L)	0.87 (0.80–0.95)	0.002
Clinical diagnosis		
GPA	1	0.002
MPA	0.72 (0.59–0.89)	
Initial induction treatment		
Daily oral cyclophosphamide	1	0.016
Pulsed cyclophosphamide	1.64 (1.10–2.45)	
Initial maintenance therapy		
AZA	1	<0.001
MMF	2.13 (1.42–3.19)	
Time to remission (per month)	1.07 (0.95–1.21)	0.279

*ANCA* anti-neutrophil cytoplasm antibodies, *AZA* azathioprine, *CI* confidence interval, *GPA* granulomatosis with polyangiitis, *MMF* mycophenolate mofetil, *MPA* microscopic polyangiitis, *MPO* myeloperoxidase, *PR3* proteinase 3

**Table 3:** The sensitivity, specificity, positive predictive value, and negative predictive value of a positive ANCA for subsequent relapse and a negative ANCA for relapse-free survival

	Positive ANCA and relapse	Negative ANCA and no relapse
Sensitivity	0.55	0.64
Specificity	0.63	0.55
Positive predictive value	0.50	0.68
Negative predictive value	0.68	0.50

*ANCA* anti-neutrophil cytoplasm antibodies

The median time to relapse was 19 (8–33) months for patients who were ANCA-positive at the switch to maintenance therapy and 23 (14–37; *p* = 0.2) months for patients who were ANCA-negative at the switch to maintenance therapy. Factors associated with an increased risk of relapse were PR3-ANCA, younger age, lower serum creatinine, GPA as a diagnosis, pulsed intravenous cyclophosphamide, and receiving MMF maintenance therapy.

**Fig. 1:**
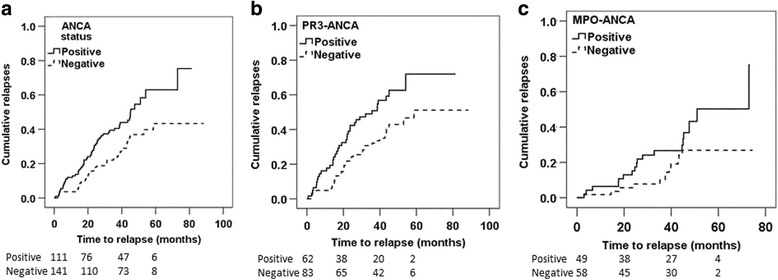
Kaplan-Meier survival curves demonstrating the effect of ANCA status at the time of switching to maintenance therapy on all patients (**a**), and patients who were initially either PR3-ANCA-positive (**b**) or MPO-ANCA-positive (**c**). The numbers remaining at risk in the ANCA-positive and ANCA-negative groups at each time point are shown below the appropriate survival curve. *ANCA* anti-neutrophil cytoplasm antibodies, *MPO*, myeloperoxidase, *PR3* proteinase 3

A graphical representation of the effect of ANCA positivity at the time of switching to maintenance therapy for all patients, and for MPO-ANCA and PR3-ANCA patients, is shown in Fig. 1.

Prior to constructing the full multivariable Cox regression survival model we tested whether there was an interaction between ANCA specificity at trial entry and ANCA status at switching to maintenance therapy. The hazard ratio for the interaction term was close to 1 and not statistically significant (HR 0.96 (0.40–2.27; *p* = 0.92)) suggesting that there was no difference in the risk conferred by MPO-ANCA and PR3-ANCA status at switching to maintenance therapy. Two multivariable Cox regression survival models were constructed: one included all covariates except ANCA status at the time of switching to maintenance therapy (not shown); the second additionally included ANCA status at the time of switching to maintenance therapy (Table 4). In both models, PR3-ANCA, pulsed intravenous cyclophosphamide induction therapy, MMF maintenance therapy, and younger age were associated with an increased risk of relapse. There was no increased risk of relapse associated with patient gender or age. In the second model, remaining ANCA-positive at the time of switching to maintenance therapy was associated with an increased risk of relapse and the addition of this variable had little effect on the hazard ratios for relapse associated with the other covariates in the model. The addition of ANCA status at switching to maintenance therapy improved the fit of the model from a pseudo *R* ^2^ value of 0.24 to 0.27.

**Table 4:** Multivariable Cox regression survival analysis of factors associated with risk of relapse

Variable	Hazard ratio (95% CI)	*p*
ANCA status at switch to maintenance therapy		
ANCA-positive	1	0.026
ANCA-negative	0.63 (0.42–0.95)	
ANCA specificity at trial entry		
MPO-ANCA	1	0.005
PR3-ANCA	1.87 (1.21–2.89)	
Initial induction treatment		
Daily oral cyclophosphamide	1	0.045
Pulsed cyclophosphamide	1.52 (1.01–2.29)	
Creatinine at entry (per 50 μmol/L)	0.89 (0.83–0.97)	0.004
Initial maintenance therapy		
AZA	1	0.002
MMF	2.08 (1.38–3.13)	
Age (per decade)	0.88 (0.76–1.01)	0.065
Gender	0.98 (0.65–1.49)	0.93
Time to remission	1.0 (0.87–1.15)	0.97

*ANCA* anti-neutrophil cytoplasm antibodies, *AZA* azathioprine, *CI* confidence interval, *MMF* mycophenolate mofetil, *MPO* myeloperoxidase, *PR3* proteinase 3

## Discussion

Previous studies have consistently demonstrated a higher risk of relapse for patients with PR3-ANCA (or cytoplasmic ANCA (cANCA)) at diagnosis compared to MPO-ANCA (or perinuclear ANCA) [17, 18, 20–22]. In this study we addressed the issue of whether persistent ANCA at the time of switching to maintenance therapy was associated with an increased risk of relapse. We demonstrated that persistent ANCA at the switch to maintenance therapy is associated with an increased risk of relapse. In multivariable analysis, this association remained significant after adjusting for initial cyclophosphamide therapy regimen, maintenance therapy, age, and renal function. When we compared two multivariable models of the risk of relapse, the model including ANCA status at the time of switching to maintenance therapy gave a better fit to the observed risk of relapse than the model not including ANCA status at the time of switching to maintenance therapy.

The few studies that have previously examined this issue were limited by smaller sample size and analyses that were not adjusted for other known risk factors for relapse. These studies suggested that persistent cANCA by indirect immunofluorescence (IIF) was associated with an increased risk of relapse compared to patients in whom ANCA re-occurred following the switch to maintenance therapy or who were persistently negative in remission [7, 23]. One recent underpowered retrospective study compared the relapse rate of patients who were cANCA-positive or cANCA-negative at the time of remission and found no significant difference in relapse risk [24]. The significance of persistent MPO-ANCA at the time of switch to maintenance therapy was investigated in 62 Japanese patients with MPA and was not found to be associated with an increased risk of relapse, although several studies have reported an increased risk of relapse associated with an increase in MPO-ANCA titre once in remission [9, 25]. Rituximab is now increasingly being used for both remission induction and maintenance in AAV. Although several trials have reported on re-occurrence of ANCA after initial remission induction as a risk factor for relapse, none have reported on the ANCA status at the time of starting maintenance therapy as a risk factor for relapse [26–28]. We tested the overall effect of remaining ANCA-positive at switching to maintenance therapy and also looked for evidence of an interaction between ANCA specificity and ANCA status at switching. There was no evidence of an interaction between these two variables, suggesting that the increased risk of relapse observed in patients remaining ANCA-positive was true for both MPO-ANCA- and PR3-ANCA-positive patients (Fig. 1).

Several factors that are recorded during routine clinical practice have now been identified that associate with an increased risk of relapse in patients with AAV: PR3-ANCA at diagnosis, persistent ANCA at switch to maintenance therapy, better renal function, younger age, pulsed cyclophosphamide for remission induction therapy, MMF for maintenance therapy, corticosteroid withdrawal, and cardiovascular system involvement [11, 12, 17, 20, 29]. Currently there is no clear evidence base to support decision making when considering maintenance or discontinuation of immunosuppression once a patient has been in remission for a period of time, although a recent meta-analysis suggested that patients should receive at least 18 months of corticosteroids to reduce the risk of subsequent relapse [29]. This study provides additional clinical information that can inform the discussion with an individual patient about the risk of relapse when planning maintenance immunosuppression therapy, although it is not possible to conclusively identify those patients who will or will not subsequently relapse—only those with a greater or lesser risk of relapse.

It is interesting to note that the patients who remained ANCA-positive at switching to maintenance therapy took longer to achieve remission than the patients who had become ANCA-negative. In the absence of hard objective measures of disease activity for AAV it is not possible to determine whether this is a real association or whether, because the physicians managing patients in the original trials were not blinded to treatment or ANCA status, knowledge of these factors influenced a physician’s judgement as to when patients entered remission and were switched to maintenance therapy. The same factors could also potentially influence the physician’s judgement as to whether symptoms represented relapse.

Patients in the CYCLOPS trial received cyclophosphamide for a longer period than the patients in the IMPROVE trial, and several CYCLOPS patients who were ANCA-positive at remission became ANCA-negative by the time of switching to maintenance therapy. Due to the small numbers of patients affected it is not possible to determine whether the additional cyclophosphamide received had a significant effect on the risk of relapse.

We are not able to determine from this study the role that ANCA plays in the development of relapse. We do not know how many of the patients who subsequently relapsed remained persistently ANCA-positive and how many of the patients who relapsed were ANCA-positive at the time of relapse. It is interesting to speculate whether ANCA positivity at the time of switching to maintenance therapy indicates a direct pathogenic role for ANCA in the re-occurrence of disease activity or whether it is a biomarker for continued lack of tolerance against the autoantigens and immunological resistance to treatment.

This study is limited in that the data are derived from two clinical trials. These studies were not designed to investigate whether remaining ANCA-positive at time of disease remission increases the risk of disease relapse. We also do not have data on how long the patients in this cohort remained on maintenance immunosuppressive therapy nor can we investigate the effect of cumulative immunosuppression and corticosteroids on relapse risk. However, it provides additional evidence that allows better relapse-risk assessment for the individual patient. It is larger than previous studies which have also suggested that ANCA positivity at the time of remission increases relapse risk and has demonstrated that this is true in multivariable analysis.

It is also important to point out that patients included in this study all received cyclophosphamide induction therapy and all but four received azathioprine or MMF maintenance therapy; thus, these results may not be applicable to patients who have received rituximab or additional plasma exchange as part of their remission induction regime.

## Conclusions

The findings of this study provide useful information for the clinician to help stratify patients for their future risk of relapse. Further work is needed in understanding why persistent ANCA at the time of switching is associated with an increased risk of relapse and whether more detailed immunophenotyping of these patients may reveal new therapeutic targets and improved understanding of the mechanisms of AAV disease. In future this may allow us to develop trials specifically targeting new or additional therapy at patients at high risk of relapse to improve outcomes.

## Abbreviations

AAV: Anti-neutrophil cytoplasm antibody-associated vasculitis
ANCA: Anti-neutrophil cytoplasm antibodies
BVAS: Birmingham Vasculitis Activity Score
cANCA: Cytoplasmic anti-neutrophil cytoplasm antibodies
CI: Confidence interval
CRP: C-reactive protein
CYCLOPS: Pulse versus continuous cyclophosphamide for induction of remission in ANCA-associated vasculitides
ELISA: Enzyme-linked immunosorbent assay
EUVAS: European Vasculitis Society
GPA: Granulomatosis with polyangiitis
HR: Hazard ratio
IIF: Indirect immunofluorescence
IMPROVE: Mycophenolate mofetil versus azathioprine for maintenance therapy in ANCA associated systemic vasculitis
MMF: Mycophenolate mofetil
MPA: Microscopic polyangiitis
MPO: Myeloperoxidase
PR3: Proteinase 3
